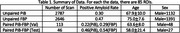# Harmonizing florbetapir and PiB PET measurements of cortical A plaque burden using unpaired regions‐of‐interest features

**DOI:** 10.1002/alz70856_102162

**Published:** 2025-12-25

**Authors:** Yiming Che, Ziqi Guo, Jay Shah, Ji Luo, Javad Sohankar, Eric M. Reiman, Yi Su, Teresa Wu

**Affiliations:** ^1^ Arizona State University, Tempe, AZ, USA; ^2^ ASU‐Mayo Center for Innovative Imaging, Tempe, AZ, USA; ^3^ Binghamton University, Binghamton, NY, USA; ^4^ Banner Alzheimer's Institute, Phoenix, AZ, USA

## Abstract

**Background:**

, we propose a Cycle‐GAN based harmonization model which eliminates the need of paired data for model training. Only a small fraction of paired data are required for model selection. By utilizing a larger number of unpaired data, it is expected to have better generality compared to using only a small number of paired data.

**Method:**

Cycle‐GAN model is adopted for unpaired harmonization in our study due to its promising performance in various unpaired tasks. Even though it is designed for image data, we modify the generator and discriminator by using multilayer perceptron (MLP) with skip‐connection for our tabular data. The model is trained on unpaired data. During the training, we utilize a small fraction of paired validation data for model selection. we first generate the translated PiB ROIs from the corresponding FBP ROIs. Then, we calculate mean‐cortical SUVR (mcSUVR) for both translated PiB ROIs and the corresponding paired PiB ROIs for Pearson correlation. The model with highest Pearson correlation is selected for further testing.

**Result:**

We summarize our data in Tab. 1. The selected model achieved testing Pearson correlation between the mcSUVR calculated from translated PiB data and the corresponding testing PiB data (85 ROIs) compared to baseline calculated from paired FBP and PiB data. According to the Steiger's Z test, our method shows statistically significant improvement (*p* <0.0001) compared to the baseline. Besides, we also tried to include extra CL feature and two demographic features (age and sex) and we achieved and respectively.

**Conclusion:**

An unpaired harmonization model based on Cycle‐GAN was developed. It only requires unpaired data for training and a small fraction of paired data for model selection. We achieved promising harmonization results on PiB and FBP measurements of cortical A.